# Therapeutic Potential of Sodium-glucose Co-transporter-2 Inhibitors and Glucagon-like Peptide-1 Receptor Agonists for Patients with Acute Coronary Syndrome: A Review of Clinical Evidence

**DOI:** 10.2174/0113816128304097240529053538

**Published:** 2024-06-20

**Authors:** Paschalis Karakasis, Dimitrios Patoulias, George Kassimis, Theocharis Koufakis, Aleksandra Klisic, Michael Doumas, Nikolaos Fragakis, Manfredi Rizzo

**Affiliations:** 1 Second Department of Cardiology, General Hospital “Hippokration”, Aristotle University of Thessaloniki, Thessaloniki, Greece;; 2 Second Propedeutic Department of Internal Medicine, General Hospital “Hippokration”, Aristotle University of Thessaloniki, Thessaloniki, Greece;; 3 Faculty of Medicine, Primary Health Care Center, University of Montenegro, Podgorica, Montenegro;; 4 Department of Health Promotion, Mother and Child Care (Promise), School of Medicine, Internal Medicine and Medical Specialties, University of Palermo, Palermo, Italy

**Keywords:** Cardiovascular disease, acute coronary syndrome, SGLT-2 inhibitor, GLP-1 receptor agonist, diabetes mellitus, drug

## Abstract

Atherosclerotic Cardiovascular Disease (ASCVD) is still one of the leading causes of death globally, with Coronary Artery Disease (CAD) being the most prevalent form of ASCVD. Patients with type 2 Diabetes Mellitus (DM) experience an increased risk for ASCVD during the disease course, with CAD being the most common cause of death among affected individuals, resulting in shorter life expectancy and increased morbidity among survivors. Recently, 2 novel classes of anti-diabetic drugs, namely Sodium-Glucose Co-Transporter-2 (SGLT-2) inhibitors and Glucagon-Like Peptide-1 (GLP-1) receptor agonists, have shown impressive cardio-renal benefits for patients with type 2 DM, while they might decrease cardio-renal risk even in the absence of baseline DM. However, there is no evidence to date regarding their safety and efficacy in the setting of an acute coronary syndrome (ACS) event, regardless of concomitant DM. This study aims to provide a detailed, updated presentation of currently available clinical evidence concerning the potential role of SGLT-2 inhibitors and GLP-1 receptor agonists in the setting of an ACS, and to highlight whether those drug classes could be utilized as adjuncts to standard-of-care treatment in this specific patient population, along with a presentation of the potential short- and long-term cardiovascular benefits.

## 1 INTRODUCTION

Coronary Artery Disease (CAD) still remains the major cause of cardiovascular death globally, despite the fact that there is significant progress in early diagnosis and prompt therapeutic intervention in high-risk patients population [1]. According to the most recent guidelines by the European Society of Cardiology (ESC), Acute Coronary Syndrome (ACS) should be considered a spectrum encompassing both Non-ST-Elevation (NSTE)-ACS and ST-Elevation Myocardial Infarction (STEMI) [2]. Of note, approximately one-fifth of patients suffering an ACS will experience cardiovascular death within the first year after the event, while one-fifth of patients will also be re-hospitalized within the same time period due to related cardiovascular complications [3]. In addition, the 5-year event rate for non-fatal Acute Myocardial Infarction (AMI), non-fatal stroke, or cardiovascular death for patients discharged after an ACS is 33.4%, according to evidence retrieved from a large cohort from the United States, with the highest event rate being observed within the first year after initial hospitalization [4].

Patients with concomitant Diabetes Mellitus (DM) have an excess risk for ACS compared to individuals without DM at baseline, with the corresponding risk being significantly higher for women compared to men [5]. In addition, patients with ACS and concomitant DM undergoing Percutaneous Coronary Intervention (PCI) have significantly higher risk for both short- and long-term cardiovascular outcomes, including cardiovascular death, compared to patients with ACS but without DM that undergo PCI [6]. On the other hand, patients with ACS without baseline DM often develop stress hyperglycemia in this context, sometimes in the context of unknown co-existing DM [7]. It has been recently documented that patients experiencing an AMI with the development of stress hyperglycemia have a significantly higher risk for major adverse cardiovascular events and in-hospital all-cause death, regardless of the presence of DM at baseline, underlining the importance of stress hyperglycemia as a prognostic marker in ACS [8].

Two classes of anti-diabetic drugs, namely Sodium-Glucose co-Transporter-2 (SGLT-2) inhibitors and Glucagon-Like Peptide-1 (GLP-1) receptor agonists, have attracted scientific interest during the last decade due to their impressive cardiovascular beneficial effects in patients with DM [9-12]. Their magnificent cardio-protective effects have led to a major revision of corresponding treatment algorithms over the last years, prioritizing the use of those two novel drug classes for patients with type 2 DM and cardiovascular co-morbidities [13-15]. Indeed, the use of SGLT-2 inhibitors may be prioritized for patients with concomitant heart failure (HF), whereas the use of GLP-1 receptor agonists may be reserved as a better treatment option for those patients having a background of Atherosclerotic Cardiovascular Disease (ASCVD) [16]. In addition, SGLT-2 inhibitor/GLP-1 receptor agonist combination regimens may confer greater cardiovascular risk reduction for patients with type 2 DM without ASCVD or HF at baseline. Thus, their use may be prioritized in the setting of primary prevention [17]. It has also been shown that SGLT-2 inhibitors provide significant cardio-renal benefits for specific patients populations, mainly those with HF or chronic kidney disease, even without concomitant DM [18, 19], whereas recent evidence also suggests that GLP-1 receptor agonists might also confer significant cardiovascular risk reduction in patients without DM at baseline, such as those being overweight or obese [20]. Thus, the question that inevitably arises is whether those two drug classes can have a place in the treatment of an ACS, as an adjunct therapeutic intervention, even in patients without pre-existing DM, and if they can confer any cardiovascular benefit for this specific patient population, both in the short- and long-term.

**Fig. (1):**
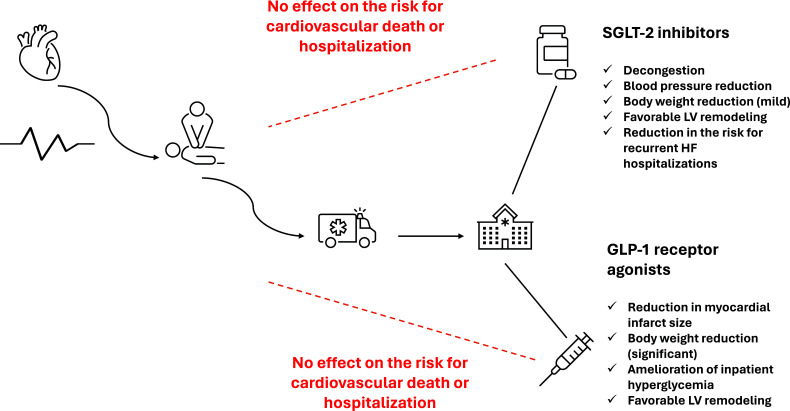
SGLT-2 inhibitors and GLP-1 receptor agonists in the setting of acute coronary syndrome.

The present review aims to present currently available evidence regarding the cardiovascular efficacy and safety of SGLT-2 inhibitors and GLP-1 receptor agonists in the setting of ACS affecting patients with and without concomitant DM (Fig. **1**).

## 2 CARDIOVASCULAR BENEFITS OF SGLT-2 INHIBITORS AND GLP-1 RECEPTOR AGONISTS: A MECHANISTIC POINT OF VIEW

There is an enormous number of preclinical and clinical studies addressing the major topic of main pathophysiologic mechanisms affected by both drug classes, which could be implicated in their established cardio-renal benefits. Several state-of-the-art review articles published over the last years have summarized those mechanisms, trying to explain -to some extent- the impressive and attractive cardiovascular effects of SGLT-2 inhibitors and GLP-1 receptor agonists.

Concerning SGLT-2 inhibitors, the primary mechanism of action is that of inhibition of renal glucose reabsorption by blocking the SGLT2 cotransporters in the proximal tubules and causing glucosuria, thus inducing glucosuria and osmotic diuresis, along with natriuresis. This results in blood pressure reduction, blood glucose reduction, and mild body weight loss [21]. Mechanisms implicated into the cardiovascular benefits of this class include improved heart energy metabolism *via* shifting to ketogenesis, amelioration of NOD-like receptor protein 3 (NLRP3) inflammasome activity, improvement in cardiac remodeling, improvement in autophagy and lysosomal degradation, amelioration of ischemia/reperfusion injury and inhibition of Na^+^/H^+^ exchange [22-25]. Other mechanisms that have been implicated in cardiovascular benefits seen with this class include reduction in arterial stiffness, improvement in endothelial function, reduction in oxidative stress, suppression of sympathetic nervous system activity, and reduction in epicardial fat mass [21-25]. Improvement in renal function, through the reduction in intraglomerular pressure, amelioration of renal hyperfiltration, and reduction in albuminuria and proteinuria, also contributes to the cardiovascular benefits of SGLT-2 inhibitors in different patient’ populations [22-25].

GLP-1 receptor agonists represent another wonderful class of drugs, primarily developed for the treatment of DM, but with a significant number of favorable pleiotropic effects in terms of cardiovascular protection. The main mechanisms of action with established cardiovascular benefit include significant body weight loss (significantly greater than that observed with SGLT-2 inhibitors) and reduction in plasma glucose levels without increasing the risk for hypoglycemia [26]. Although they do not exert similar effects with SGLT-2 inhibitors in terms of plasma volume contraction, they have been shown to suppress inflammation, ameliorate ischemia/reperfusion injury, enhance a favorable cardiac remodeling, and reduce epicardial fat mass *via* their favorable effect on ectopic fat deposits [27, 28]. There is also -mainly preclinical- evidence suggesting that GLP-1 receptor agonists exert anti-atherosclerotic and vasodilatory effects in epicardial coronary arteries, resulting in plaque stability, prevention of plaque rupture, and increase in myocardial blood flow [29, 30]. However, despite the fact that there is a significant amount of clinical evidence suggesting a net cardiovascular benefit with GLP-1 receptor agonists use in DM, mainly driven by the reduction in ASCVD events, underlying mechanisms are not yet fully understood.

## 3 SGLT-2 INHIBITORS AND THE RISK FOR CORONARY ARTERY DISEASE

A formerly published meta-analysis of 35 Randomized Controlled Trials (RCTs) in a total of 34,987 patients with type 2 DM showed that treatment with SGLT-2 inhibitors *versus* control resulted in a significant reduction by 15% in the odds for non-fatal AMI, whereas they did not confer any significant benefit on the occurrence of unstable angina [31]. Of note, treatment with SGLT-2 inhibitors resulted in a significant decrease in the odds of all-cause death by 21% and in the odds of Major Adverse Cardiovascular Events (MACEs) by 20%, compared to the control group [31]. Conflicting evidence was provided by another, more recent meta-analysis of 21 RCTs in a total of 56,064 patients with or without type 2 DM, which demonstrated a non-significant effect of SGLT-2 inhibitor treatment on the risk for AMI, unstable angina, or angina pectoris [32].

A dedicated meta-analysis of the hallmark Ccardiovascular Outcome Trials (CVOTs) with SGLT-2 inhibitors in a total of 46,969 patients with concomitant type 2 DM, two-thirds of whom had pre-existing ASCVD, documented that treatment with SGLT-2 inhibitors *versus* placebo conferred a significant risk reduction by 9% regarding the occurrence of AMI; of importance, this effect was significant for those patients having pre-existing ASCVD, with a relative risk reduction of 10%, while, no significant effect on the risk for AMI was documented for patients without prior ASCVD [33].

Therefore, it seems that especially for patients with concomitant type 2 DM, treatment with SGLT-2 inhibitors may decrease the risk for subsequent AMI, while it might have no or minimal effect on the risk for other forms of CAD, mainly unstable angina.

## 4 SGLT-2 INHIBITORS IN THE SETTING OF ACUTE CORONARY SYNDROME

### 4.1 Evidence from RCTs

Earlier in 2023, we welcomed the results of the first dedicated CVOT with SGLT-2 inhibitors in patients presenting with ACS, the DAPA-MI trial. In short, trialists of DAPA-MI enrolled 4,017 clinically stable patients with a recent AMI (both STEMI and NSTEMI), that occurred within the previous 10 days before enrollment and impaired Left Ventricular (LV) systolic function [34]. None of the enrolled subjects could have pre-existing DM or HF, according to the pre-specified inclusion and exclusion criteria [34]. Eligible patients were then randomized either to dapagliflozin 10 mg once daily or placebo, as an adjunct to standard-of-care treatment [34]. After a mean follow-up duration of 24 months, it was shown that dapagliflozin did not result in a significant effect on the risk for MACEs, all-cause death, cardiovascular death, AMI recurrence, hospitalization for HF, stroke, or the composite endpoint of cardiovascular death, hospitalization for HF or AMI recurrence [34]. However, dapagliflozin treatment resulted in a significant improvement in cardiometabolic outcomes, compared to placebo, as it led to a significant, beneficial effect on the primary composite endpoint, which consisted of all-cause death, HF hospitalization, nonfatal AMI, atrial fibrillation/flutter event, type 2 DM, New York Heart Association (NYHA) class or body weight decrease of ≥ 5%, compared to baseline [34]. In addition, dapagliflozin treatment, when compared to a placebo, resulted in a significant decrease in the risk for a new diagnosis of type 2 DM, equal to 47% [34]. Therefore, based on the results of the DAPA-MI trial, the cardiovascular benefits of SGLT-2 inhibitors in the setting of ACS appear to be limited.

Another recently published trial is The Study to Test the Effect of Empagliflozin on Hospitalization for Heart Failure and Mortality in Patients with Acute Myocardial Infarction (EMPACT-MI) [35]. EMPACT-MI trial screened 6,610 patients and randomized 6,522 of them either to empagliflozin 10 mg once daily or placebo within the first 14 days after hospital admission for AMI [36]. The mean time from AMI to randomization was 5 days, whereas only 13% of enrolled patients had a history of prior AMI, and one-third of them had pre-existing DM [36]. Of note, enrolled subjects had severely impaired LV systolic function, with a mean LV ejection fraction equal to 40%, whereas more than half of them had signs or symptoms of congestion at baseline prior to randomization [36]. The primary composite endpoint is time to the first hospitalization for HF or all-cause mortality. During a median follow-up period of 17.9 months, the occurrence of either a first hospitalization for HF or death from any cause was noted in 267 patients (8.2%) in the empagliflozin group and in 298 patients (9.1%) in the placebo group. This corresponded to incidence rates of 5.9 and 6.6 events per 100 patient-years, respectively [hazard ratio (HR) 0.90; 95% confidence interval (CI) 0.76-1.06). Regarding the individual components of the primary endpoint, a first hospitalization for HF was experienced by 118 patients (3.6%) in the empagliflozin group compared to 153 patients (4.7%) in the placebo group (HR 0.77; 95% CI 0.60-0.98). Death from any cause occurred in 169 patients (5.2%) and 178 patients (5.5%) in the empagliflozin and placebo groups, respectively (HR 0.96; 95% CI 0.78-1.19). Adverse events were consistent with the established safety profile of empagliflozin and demonstrated similar occurrence rates between the two trial groups. Secondary analyses examining the cardiometabolic effects of empagliflozin in the studied patient population are eagerly awaited.

EMpagliflozin in patients with acute myocardial infarction (EMMY) trial was another RCT that investigated the effects of empagliflozin in the setting of ACS. More specifically, trialists enrolled 476 hemodynamically stable patients at least 72 hours after undergoing a PCI for AMI and randomized them in a 1:1 ratio either to empagliflozin 10 mg once daily or matching placebo [37]. Less than one-fifth of enrolled subjects had pre-existing type 2 DM (13%) or CAD (11%) [37]. Regarding the primary endpoint, trialists demonstrated that empagliflozin, compared to placebo, resulted in a significant decrease in *N*-terminal pro-hormone of brain natriuretic peptide (NT-proBNP) levels over 26 weeks of treatment. In contrast, it was also shown that empagliflozin treatment had a significant beneficial effect on LV systolic function. It significantly improved structural cardiac changes, compared to baseline, as indicated by significant improvements in LV ejection fraction and LV end-systolic and end-diastolic volumes [37]. A significant decrease in body weight was also seen with empagliflozin over 26 weeks of treatment [37]. Of note, no significant difference was observed regarding all-cause death, cardiovascular death, and hospitalization for HF or MACEs, mainly attributed to the scarcity of events. In a post-hoc analysis of the EMMY trial, a significant reduction in inflammatory burden, as evaluated with the change in several inflammatory biomarkers, was observed in both treatment arms; however, no significant difference between empagliflozin and placebo was shown [38]. According to another post-hoc analysis of the EMMY trial, early administration of empagliflozin, within the first 24 hours after PCI does not seem to pose any safety risk for patients with AMI, while it appears to be similarly effective in terms of improvement in LV systolic and diastolic function and reduction in NT-pro-BNP levels, compared to late initiation, after the first 48-72 hours post-PCI [39].

Finally, the EMBODY trial was another RCT in the field, which enrolled 105 patients with a recent AMI (2 weeks) and concomitant type 2 DM and randomized either to empagliflozin 10 mg once daily or placebo [40]. Less than one-fifth of enrolled participants had prior ASCVD, whereas they had a relatively short duration of type 2 DM [40]. Trialists aimed at identifying whether empagliflozin could exert a beneficial effect on cardiac sympathetic nerve activity in patients with type 2 DM after an AMI. Therefore, they set the change in heart rate variability (HRV) as the primary efficacy endpoint over 26 weeks of treatment [30]. Whereas a significant improvement in various indices of HRV, along with a significant improvement in heart rate turbulence, was shown in the empagliflozin arm, no significant difference between empagliflozin and placebo was demonstrated [40]. A significant decrease in systolic blood pressure was identified as a driving mediating mechanism for the improvement of HRV in the empagliflozin arm [40]. No serious adverse events, including cardiac events, were observed in both treatment arms [40]. In a post-hoc analysis of the EMBODY trial, trialists demonstrated that empagliflozin resulted in a significant decrease in plasma volume status and in NT-pro-BNP levels, findings suggestive of a potential benefit for patients with a recent AMI and symptoms/signs indicative of congestion [41].

### 4.2 Evidence from Observational Studies

There is a significant lack of evidence from observational studies regarding the safety and efficacy of SGLT-2 inhibitors in the setting of an ACS when administered either in-hospital or early after discharge.

A formerly published, prospective observational study enrolled 44 patients with a recent ACS and concomitant type 2 DM, who were allocated at discharge either to empagliflozin 10 or 25 mg once daily plus standard-of-care, or only standard-of-care [42]. Almost one-fourth of the enrolled participants had a prior diagnosis of CAD, whereas 75% of them underwent PCI at admission [42]. Of note, patients allocated to empagliflozin had significantly worse glycemic control, justifying the decision for empagliflozin initiation, based on the observational nature of the study [42]. After a 3- month follow-up period, researchers observed a significant reduction in LV mass index and LV diastolic function parameters (*e.g*. mitral valve peak E-wave velocity and E/e′ ratios), whereas there was no significant difference in LV global longitudinal strain and LV systolic function, as assessed by LV ejection fraction [42]. A significant correlation between the change in hematocrit and the change in average E/e’ ratio, along with a significant correlation between the change in NT-pro-BNP levels and LV mass index, was documented [42]. No safety issues or serious adverse events were reported.

Another observational study enrolling 2,814 patients with a history of type 2 DM and AMI assessed, after 1:2 propensity score matching, the effect of SGLT-2 inhibitor initiation within the first 14 days after PCI for AMI on surrogate cardiovascular endpoints of interest [43]. Researchers demonstrated that SGLT-2 inhibitor initiation after a median follow-up period of 2.1 years resulted in a significant decrease in the risk for the primary composite endpoint (all-cause death or hospitalization for HF) by 32% and in the risk for the secondary composite endpoint (all-cause death, non-fatal AMI or non-fatal ischemic stroke) by 23% [43]. A significant reduction in the risk of all-cause death by 45% and for hospitalization for HF by 24% was also shown, whereas no significant effect on the risk for non-fatal AMI recurrence or for non-fatal ischemic stroke was demonstrated [43]. No significant interaction between participants’ baseline characteristics of interest and the primary composite endpoint was documented [43].

In another retrospective observational study, including 420 patients with a recent diagnosis of CAD and concomitant type 2 DM, it was assessed whether initiation of an SGLT-2 inhibitor at discharge had a more favorable impact on surrogate cardiovascular outcomes [44]. The presentation of CAD was ACS in 44.3% of patients and chronic coronary syndrome in the rest [44]. After a mean follow-up period of 3 years, patients administered SGLT-2 inhibitors at discharge experienced a significant reduction in the risk for all-cause death by 68%, whereas no significant effect on the risk for cardiovascular death, AMI, stroke, or hospitalization for HF was shown [44]. Of note, a significant increase in the risk for unplanned revascularization in the SGLT-2 inhibitor arm was also demonstrated without assessment of the possible mediators [44].

Interesting results were generated by another retrospective observational study enrolling 198 patients with type 2 DM and stabilized AMI, who were assigned either to the SGLT-2 inhibitor plus standard-of-care arm or the standard-of-care arm alone [45]. In the SGLT-2 inhibitor arm, 78.8% of enrolled subjects were prescribed an SGLT-2 inhibitor after the AMI, while the rest were already on SGLT-2 inhibitor treatment prior to AMI manifestation [45]. After a mean follow-up period of 23.5 months, 3 patients in the SGLT-2 inhibitor arm *versus* 22 patients in the non-SGLT-2 inhibitor arm experienced rehospitalization for ACS, while 1 patient receiving SGLT-2 inhibitor *versus* 7 patients not receiving SGLT-2 inhibitors experienced sudden cardiac death [45]. However, no subgroup analysis was performed at the time of SGLT-2 inhibitor treatment initiation [45].

A limited number of studies have also assessed the impact of prior SGLT-2 inhibitor treatment on surrogate outcomes of interest among patients with type 2 DM experiencing an AMI, showing a clear benefit on cardiovascular morbidity and mortality with this drug class [46-48]. In addition, prior SGLT-2 inhibitor treatment in patients with type 2 DM and AMI was also shown to be associated with a significantly lower risk for intra-stent restenosis events [48]. Interestingly, data from the same cohort of patients suggest a beneficial effect of prior SGLT-2 inhibitor treatment on the occurrence of supraventricular and ventricular arrhythmias in patients with AMI [49]. However, the observational nature of the studies and the retrospective study design, along with the relatively long SGLT-2 inhibitor treatment duration prior to ACS development, do not permit definitive conclusions.

A summary of major endpoints assessed in both RCTs and observational studies is provided in Table **1**.

## 5 GLP-1 RECEPTOR AGONISTS AND THE RISK FOR CORONARY ARTERY DISEASE

It has been long established that GLP-1 receptor agonists provide significant cardiovascular benefits for patients with type 2 DM, especially for those with concomitant ASCVD, based upon the results of the hallmark CVOTs published during the last decade. According to a former meta-analysis of the dedicated CVOTs in the field, GLP-1 receptor agonist treatment in patients with type 2 DM has been shown to provide a significant reduction in the risk for fatal or non-fatal AMI by 9%, compared to placebo [50]. An updated meta-analysis of available CVOTs, published 2 years later, also confirmed that GLP-1 receptor agonists decrease the risk for fatal or non-fatal AMI by 10% in patients with type 2 DM, compared to placebo, demonstrating a significant treatment benefit for patients with type 2 DM being at high cardiovascular risk or having a history of ASCVD [51]. Those results have resulted in a substantial modification in the corresponding treatment algorithms of type 2 DM in clinical practice over the past few years [13, 14]; however, the exact role of GLP-1 receptor agonists in the setting of an ACS has yet to be clarified.

## 6 GLP-1 RECEPTOR AGONISTS IN THE SETTING OF ACUTE CORONARY SYNDROME

### 6.1 Evidence from RCTs

The Evaluation of Lixisenatide in Acute Coronary Syndrome (ELIXA) trial still represents the hallmark study concerning the use of GLP-1 receptor agonists in patients with ACS [52]. Trialists recruited 6,068 patients with type 2 DM and either a recent AMI or hospitalization for unstable angina within the last 6 months, who were randomized either to lixisenatide or placebo, as an adjunct to standard-of-care treatment. The mean time period from the event to randomization was about 72 days in both treatment arms [52]. Lixisenatide failed to produce a statistically significant effect on the primary composite endpoint, consisting of cardiovascular death, non-fatal stroke, non-fatal AMI, or unstable angina, while it did not also exert any significant beneficial effect on each component separately, along with the risk for hospitalization for HF and all-cause death [52]. Regarding other effects on cardio-metabolic parameters of interest, lixisenatide led to significant, but small numerically, reductions in glycemic control, body weight, and systolic blood pressure [52]. Practically, based on the results of the ELIXA trial, lixisenatide has been downgraded as a treatment option for patients with type 2 DM at high cardiovascular risk or those having established ASCVD [52].

A mechanistic trial with acute exenatide treatment in patients with STEMI undergoing primary PCI was published a decade ago [53]. A total of 172 patients with STEMI, 6% of whom had pre-existing DM, were randomized either to exenatide or normal saline continuous intravenous infusion, starting 15 minutes before primary PCI and maintained for 6 hours after the intervention [53]. Performance of cardiac magnetic resonance imaging (cMRI) 3 months later in both treatment arms demonstrated that acute exenatide treatment *versus* control resulted in significantly greater myocardial salvage index and significantly lower infarct size/area at risk ratio [53]. Left ventricular systolic function at 3 months post-intervention did not differ between the 2 treatment arms, whereas no difference in the occurrence of serious adverse events was observed [53]. A similar mechanistic trial was published at the same time, enrolling patients without previously known DM, who were admitted to the hospital with STEMI and underwent primary PCI [54]. Patients were randomized either to continuous exenatide or placebo intravenous infusion, which started just prior to the intervention and continued for 72 hours post-intervention [54]. Patients were followed and underwent cMRI 4 months post-intervention, which showed a trend towards smaller infarct size in patients treated with exenatide, although the result remained non-significant [54]. In addition, no difference in LV systolic function between the 2 study groups was shown [54].

A third, similar, mechanistic trial was published a year later, enrolling 58 patients presenting with STEMI and undergoing primary PCI, who were randomized either to exenatide or placebo, administered subcutaneously before the onset of reperfusion and continued twice daily, for the following 48 hours after PCI [55]. Performance of cMRI 1 month later revealed a significantly lower myocardial infarct size in patients assigned to exenatide compared to placebo [55]. A significant improvement in both LV systolic and diastolic function was observed in both treatment arms after 6 months [55]. Another trial was published in 2016, and it also enrolled 91 patients presenting with STEMI and undergoing primary PCI [56]. Despite promising results of the former trials, intravenous exenatide infusion for 72 hours post-intervention did not have any significant effect on myocardial infarct size, as assessed with cMRI 4 months after PCI [56]. Of note, no serious adverse events were observed in both treatment arms [56].

A limited number of RCTs have also been performed with liraglutide, a GLP-1 receptor agonist with established cardio-protective effects. In a relevant RCT enrolling 92 patients admitted with STEMI (with less than one-fifth of them having concomitant type 2 DM at baseline), it was shown that treatment with once-daily subcutaneous liraglutide, started 30 minutes prior to PCI and continued for 7 days, compared to placebo resulted in a significant improvement in LV systolic function after 3 months [57]. In addition, liraglutide treatment resulted in significantly lower fasting plasma glucose levels within hospitalization, *versus* placebo, ameliorating the negative impact of stress hyperglycemia, whereas it also produced a significant decrease in systemic inflammatory markers at 3 months of follow-up [57]. Of note, there was a tendency towards a lower incidence of no-reflow phenomenon in the liraglutide group, a finding that did not reach statistical significance [57]. No significant difference in the risk for MACE or cardiovascular death was observed between liraglutide and placebo. A post-hoc analysis of this RCT documented that inpatient treatment with liraglutide in patients with STEMI resulted in significantly higher myocardial salvage index and significantly smaller infarct size, compared to placebo, according to cMRI results performed at 3 months post-PCI [58]. Of importance, the significant difference in myocardial salvage index between the 2 treatment arms correlated negatively with the difference in fasting plasma glucose levels and in high-sensitivity C-reactive protein (hsCRP) levels, highlighting potential mechanisms which could be implicated in the cardio-protective effects of liraglutide in the acute setting of STEMI [58].

The same research team performed another relevant RCT, enrolling 210 patients with STEMI, who were allocated to a single dose of liraglutide administered subcutaneously or placebo prior to coronary angiography and PCI [59]. After 3 months, the primary endpoint, namely the prevalence of the no-reflow phenomenon, was significantly lower in the liraglutide arm than in the placebo arm [59]. In addition, acute liraglutide treatment resulted in significantly greater LV systolic function at 3 months, along with significantly lower fasting plasma glucose levels, hsCRP levels, and troponin levels in the inpatient setting [59]. No significant difference in the risk for MACE, hospitalization for HF, or cardiovascular death at 3 months post-PCI was observed between the 2 treatment groups [59]. Another RCT assessing the efficacy and safety of liraglutide in the setting of NSTE-ACS was performed by the same researchers [60]. A total of 90 patients presenting with NSTE-ACS and undergoing primary PCI, less than one-third of whom had baseline DM, were assigned either to once weekly subcutaneous liraglutide or placebo [60]. After 3 months, patients assigned to liraglutide *versus* those randomized to placebo experienced a significant improvement in LV systolic function, as assessed by LVEF and LV stroke volume, meeting the primary efficacy endpoint [60]. Liraglutide also conferred a significant reduction in fasting plasma glucose levels in the acute setting, a finding highlighting its efficacy against stress hyperglycemia [60]. Notably, researchers demonstrated that, at 3 months, those patients randomized to liraglutide in the inpatient setting had significantly lower levels of hsCRP and oxidative stress markers, especially those not having DM at baseline [60]. After a 6-month follow-up period, no significant difference in the risk for MACE, recurrent AMI, or cardiovascular death was observed between the 2 study groups [60].

Interesting results were obtained from another pilot RCT, which assessed the impact of liraglutide on glycemic variability indices in a small number of patients with type 2 DM and a recent ACS [61]. A total of 13 patients hospitalized with an ACS (9 with AMI, 4 with unstable angina), were randomized either to subcutaneous liraglutide or insulin glargine, after appropriate modification of the baseline antidiabetic treatment [61]. Whereas no significant difference between the 2 treatment arms regarding short-term GV was shown, it was demonstrated that after 12 weeks of follow-up, patients randomized to liraglutide had a more favorable GV profile with significant differences in standard deviation and coefficient variation, despite the non-significant difference at the level of glycemic control [61]. No remarkable safety issues in either treatment arms were noted [61]. Those results are considered as important, despite the small sample size, based on the prognostic significance of GV in patients with type 2 DM [62].

### 6.2 Evidence from Observational Studies

There are only a few observational studies assessing the efficacy of GLP-1 receptor agonists in patients with ACS with or without DM. In a prospective observational study, 8 patients with type 2 DM and a recent (<2 weeks) hospitalization due to STEMI, undergoing PCI, were initiated on liraglutide treatment as an adjunct to standard of care [63]. After 24 weeks of treatment, the researcher demonstrated a significant improvement in glucose-metabolic parameters, including body weight, blood pressure, and glycated hemoglobin levels; however, no significant difference in LV remodeling indices was noted, whereas no significant change in inflammatory and oxidative stress markers was also shown [63].

In another retrospective observational study enrolling 15 patients with a recent ACS and concomitant type 2 DM, undergoing primary PCI and followed-up for 6 months with conduction of cMRI, it was demonstrated that treatment with liraglutide at discharge, compared to non-GLP-1 receptor agonist treatment, was associated with significantly lower LV mass index, while no difference in other LV remodeling indices was observed [64].

A summary of major endpoints assessed in both RCTs and observational studies is provided in Table **2**.

## CONCLUSION

It is well-established that both GLP-1 receptor agonists and SGLT-2 inhibitors are the most prominent antidiabetic drug classes that have changed the therapeutic management of type 2 DM over the last years, mainly due to their multiple pleiotropic effects, despite the fact that their mechanisms of action have not been fully elucidated yet [65-70].

Currently, available evidence concerning the efficacy of SGLT-2 inhibitors and GLP-1 receptor agonists in the setting of an ACS is rather conflicting. On the one hand, SGLT-2 inhibitors appear to improve LV systolic and diastolic function and produce decongestion for patients with a recent ACS, along with a significant improvement in several cardiometabolic parameters of interest, including glycemia, body weight, and blood pressure. However, as clearly shown in the recently published DAPA-MI trial, they do not exert any beneficial effect on surrogate cardiovascular endpoints when administered in the acute setting in this population. On the other hand, GLP-1 receptor agonists might decrease myocardial infarct size and improve LV remodeling indices after an AMI, although evidence is rather conflicting. In addition, they also exert significant benefits in cardiometabolic parameters, even at greater levels than SGLT-2 inhibitors. However, there are no dedicated CVOTs with GLP-1 receptor agonists in the setting of ACS, therefore, no solid evidence regarding their true cardiovascular efficacy on surrogate endpoints can be generated.

Reasons, why some beneficial effects exerted by both drug classes were not seen in clinical studies, including study design (RCTs *versus* observational studies), sample size, selection of prespecified outcomes, differences in baseline characteristics of enrolled subjects across the various studies (both RCTs and observational) and the selection of various SGLT-2 inhibitors and GLP-1 receptor agonists (for example, semaglutide, liraglutide, and dulaglutide exert strong cardiovascular benefits, whereas exenatide and lixisenatide not). Future studies are warranted to reveal the relationship between speculated mechanisms and clinical outcomes in patients with ACS.

Additional research is needed to investigate the potential effects of novel glucose-lowering agents in specific subpopulations with Acute Coronary Syndrome (ACS). Firstly, the enrolled population in the trials was predominantly managed invasively through primary percutaneous coronary intervention. However, in real- world practice, many patients, particularly those who are frail, are managed conservatively with optimal medical therapy [71]. Therefore, further elucidation is needed to determine whether this specific patient population would benefit more from enhanced medical treatment. Secondly, considering the demonstrated anti-inflammatory properties of SGLT-2 inhibitors and GLP-1 receptor agonists, ACS patients with a high inflammatory burden are expected to potentially derive greater benefit. Thirdly, unstable angina, another component of ACS often neglected, was excluded from major relevant trials and should, therefore, be explored in future studies. Fourthly, novel therapeutic approaches targeting specific underlying mechanisms of myocardial infarction with non-obstructive coronary arteries (MINOCA), such as endothelial dysfunction or inflammation, have shown promising results in preclinical studies [72]. Since SGLT-2 inhibitors and GLP-1 receptor agonists have demonstrated beneficial effects on both endothelial dysfunction and inflammation, their utilization in MINOCA patients seems promising and warrants further investigation in specifically designed studies.

Despite their clear cardiovascular benefits in the broad population of type 2 DM, and more recently in other patients’ populations, regardless of DM status at baseline, existing evidence does not support the use of SGLT-2 inhibitors and GLP-1 receptors in the setting of an ACS. Large-scale, well-designed RCTs are required to definitely answer this interesting research question, which can expand the use of both drug classes in clinical practice.

## Figures and Tables

**Table 1:** Summary of the available studies assessing the effects of SGLT-2 inhibitors in patients with acute coronary syndrome, administered either in-hospital or early after discharge.

**Study/Study Design**	**SGLT-2 Inhibitor**	**Study Population**	**Cardiovascular Mortality**	**All-cause Mortality**	**Hospitalization for Heart Failure**	**Major Adverse Cardiovascular Events**	**Other Cardio-metabolic Benefits of SGLT-2 Inhibitor Treatment**
James *et al*. [34] RCT	Dapagliflozin	4,017	HR 1.15 (95% CI; 0.66-2.01)	HR 1.22 (95% CI; 0.77-1.92)	HR 0.83 (95% CI; 0.50-1.39)	HR 0.94 (95% CI; 0.67-1.31)	• Significant reduction in body weight. • Significant reduction in the risk for new diagnosis of type 2 DM.
von Lewinski *et al*. [37] RCT	Empagliflozin	476	Empagliflozin: 2/237	Empagliflozin: 3/237	Empagliflozin: 3/237	Empagliflozin: 2/237	• Significant reduction in body weight. • Significant decrease in NT-pro-BNP levels. • Significant improvement in LV systolic and diastolic function.
			Placebo: 0/239	Placebo: 0/239	Placebo: 4/239	Placebo: 5/239	
Bulter *et al*. [36] RCT	Empagliflozin	6522	HR 1.03 (95% CI; 0.81-1.31)	HR 0.96; (95% CI; 0.78-1.19)	HR 0.77 (95% CI; 0.60-0.98)	Not reported	• The adverse events observed, including ketoacidosis and hypoglycemia events, were consistent with the established safety profile of empagliflozin and exhibited comparable occurrence rates between the two trial groups.
Shimizu *et al*. [40] RCT	Empagliflozin	96	No events reported	No events reported	No events reported	No events reported	• Improvement in indices of HRV. • Significant reduction in body weight, SBP and uric acid levels. • Significant decrease in PVS. • Significant decrease in NT-pro-BNP levels.
Lan *et al*. [42] Observational	Empagliflozin	44	No events reported	No events reported	No events reported	No events reported	• Significant reduction in LV mass index. • Significant improvement in LV diastolic function. • Significant reduction in HbA1c and increase in hematocrit levels.
Kwon *et al*. [43] Observational	Dapagliflozin Empagliflozin Ipragliflozin	2,814	Not reported	HR 0.55 (95% CI; 0.37-0.80)	HR 0.76 (95% CI; 0.56-0.98)	HR 0.77 (95% CI; 0.60-0.99)	Not reported
Chipayo-Gonzales *et al*. [44] Observational	Dapagliflozin Empagliflozin Canagliflozin	420	HR 0.52 (95% CI; 0.19-1.44)	HR 0.32 (95% CI; 0.12-0.81)	HR 0.85 (95% CI; 0.36-2.02)	AMI: HR 1.55 (95% CI; 0.66-3.66)	Not reported
						Stroke: HR 0.57 (95% CI; 0.08-4.02)	
Chang *et al*. [45] Observational	Empagliflozin	-	Empagliflozin: 1/66	Not reported	Not reported	Not reported	Not reported
		198	Control: 7/132				

**Abbreviations:** SGLT-2: sodium-glucose co-transporter-2; DM: diabetes mellitus; HRV: heart rate variability; LV: left ventricular; SBP: systolic blood pressure; NT-pro-BNP: N-terminal pro b-type natriuretic peptide; PVS: plasma volume status; RCT: randomized controlled trial; HR: hazard ratio.

**Table 2:** Summary of the available studies assessing the effects of GLP-1 receptor agonists in patients with acute coronary syndrome, administered either in-hospital or early after discharge.

**Study/Study Design**	**GLP-1 Receptor Agonist**	**Study Population**	**Cardiovascular Mortality**	**All-cause Mortality**	**Hospitalization for Heart Failure**	**Major Adverse Cardiovascular Events**	**Other Cardio-metabolic Benefits of GLP-1 Receptor Agonist Treatment**
Pfeffer *et al*. [52] RCT	Lixisenatide	6,068	HR 0.98 (95% CI; 0.78-1.22)	HR 0.94 (95% CI; 0.78-1.13)	HR 0.96 (95% CI; 0.75-1.23)	HR 1.02 (95% CI; 0.89-1.17)	(1) Significant reduction in HbA1c, body weight and systolic blood pressure.
Lønborg *et al*. [53] RCT	Exenatide	172	Exenatide: 2/85	Not reported	Not reported	AMI Exenatide: 1/85 Placebo: 0/87	(1) Significant reduction in myocardial infarct size.
			Placebo: 2/87			Stroke Exenatide: 1/85 Placebo: 1/87	
Bernink *et al*. [54] RCT	Exenatide	43	No events reported	No events reported	No events reported	No events reported	(1) Non-significant reduction in myocardial infarct size.
Woo *et al*. [55] RCT	Exenatide	58	No events reported	No events reported	No events reported	No events reported	(1) Significant reduction in myocardial infarct size. (2) Significant improvement in LV systolic and diastolic function.
Roos *et al*. [56] RCT	Exenatide	91	No events reported	No events reported	No events reported	No events reported	(1) Non-significant reduction in myocardial infarct size.
Chen *et al*. [57, 58] RCT	Liraglutide	92	Liraglutide: 1/45	Not reported	Not reported	Liraglutide: 6/45	(1) Significant improvement in LV systolic function. (2) Significantly higher myocardial salvage index with liraglutide. (3) Significant reduction in inflammatory markers.
			Placebo: 2/47			Placebo: 12/47	
Chen *et al*. [59] RCT	Liraglutide	210	Not reported	Not reported	Not reported	Liraglutide: 8/105	(1) Significant improvement in LV systolic function. (2) Significantly lower prevalence of no-reflow phenomenon. (3) Significant reduction in stress hyperglycemia and inflammatory markers in the inpatient setting.
						Placebo: 16/105	
Chen *et al*. [60] RCT	Liraglutide	90	Liraglutide: 0/45	Not reported	Not reported	Liraglutide: 5/45	(1) Significant improvement in LV systolic function. (2) Significant amelioration of inpatient stress hyperglycemia. (3) Significant decrease in inflammatory and oxidative stress markers.
			Placebo: 1/45			Placebo: 9/45	
Del Olmo-García *et al*. [61] RCT	Liraglutide	13	No events reported	No events reported	No events reported	Liraglutide: 0/5	(1) Significant improvement in glycemic variability. (2) Significant reduction in prandial insulin dosage required during hospitalization.
						Insulin glargine: 1/8	
Kajiwara *et al*. [63] Observational	Liraglutide	8	No events reported	No events reported	No events reported	No events reported	(1) Significant improvement in body weight, blood pressure and HbA1c levels.
Nozue *et al*. [64] Observational	Liraglutide	15	Not reported	Not reported	Not reported	No events reported	(1) Significant reduction in LV mass index. (2) Significant reduction in LDL-cholesterol.

**Abbreviations:** GLP-1: glucagon-like peptide-1; HR: hazard ratio; AMI: acute myocardial infarction; RCT: randomized controlled trial; HbA1c: glycated hemoglobin; LDL: low-density lipoprotein.

## LIST OF ABBREVIATIONS

ACS: Acute Coronary Syndrome
CAD: Coronary Artery Disease
DM: Diabetes Mellitus
ESC: European Society of Cardiology
